# The impact of the COVID-19 pandemic restrictions on the health care utilization of cancer patients

**DOI:** 10.1186/s12885-023-10945-9

**Published:** 2023-05-15

**Authors:** Nico Schneider, Bernhard Strauss, Jutta Hübner, Christian Keinki, Florian Brandt, Sabine Rubai, Uwe Altmann

**Affiliations:** 1 Institute of Psychosocial Medicine, Psychotherapy and Psychoonocology, Jena University Hospital, Stoystrasse 3, Jena, D-07740 Germany; 2 Department of Haematology and Medical Oncology, Jena University Hospital, Am Klinikum 1, Jena, D-07747 Germany; 3 The German Cancer Society, Kuno-Fischer-Strasse 8, Berlin, D-14057 Germany; 4 IKK Südwest, Europaallee 3-4, Saarbrücken, D-66113 Germany; 5 Saarland Cancer Society, Bruchwiesenstrasse 15, Saarbrücken, D-66111 Germany; 6 Department of Psychology, MSB Medical School Berlin GmbH, Berlin, Germany

**Keywords:** Cancer, COVID-19, Psycho-social counseling, Online knowledge database, Patient navigator

## Abstract

**Background:**

COVID-19 has impacted both society and medical care. While Germany entered the first lockdown in spring 2020, the PIKKO study (Patient information, communication and competence empowerment in oncology) was still active. The intervention modules, patient navigator (PN), services of the Saarland Cancer Society (SCS), psycho-social counseling and different courses, and online knowledge database (ODB) continued to be offered, but in an adapted form. It was the aim of this supplementary survey to identify the restrictions and burdens of the pandemic containment strategies on the PIKKO patients and thus on the PIKKO study itself. Furthermore, this work shows how the PIKKO modules were used during the lockdown.

**Methods:**

All patients in the PIKKO intervention group (IG) were invited to complete a questionnaire, n = 503. Furthermore, utilization of the SCS and log files of the ODB were analyzed. For socio-demographic data and contacts with the PN, data from the regular PIKKO surveys were used. In addition to descriptive statistics, chi²-tests, F-tests and linear regression analyses were performed.

**Results:**

356 patients participated in this supplemental survey. 37.6% reported restrictions. “Restrictions on accompanying persons”, “ban on visits to the wards” and “protective mouth-nose-mask” were reported as the greatest burdens. 39.0% expressed fears that the restrictions would have an impact on the course of their disease. Linear regression analyses showed differences in feelings of burden among age groups (more among < 60-year-olds), gender (more among women), children in the household (more with children), and preexisting financial stress (more with financial worries). In April 2020, there was more patient contact with PNs by phone, more SCS psycho-social counseling by phone, adapted SCS course offering, but with significantly fewer participants, and high activity on the ODB.

**Conclusion:**

Cancer patients in the IG reported restrictions from the pandemic containment strategies and feared an impact on their recovery. However, whether a burden is perceived as heavy depends more on gender, age, or pre-existing burdens than on whether the lockdown affects PIKKO or not. The utilization of counseling, courses or the ODB despite lockdown shows the need for such services, especially in times of crisis.

**Trial registration:**

This study was retrospectively registered in the German Clinical Trial Register under DRKS00016703 (21 Feb 2019, retrospectively registered). https://www.drks.de/drks_web/navigate.do?navigationId=trial.HTML&TRIAL_ID=DRKS00016703.

**Supplementary Information:**

The online version contains supplementary material available at 10.1186/s12885-023-10945-9.

## Background

Since the beginning of 2020, COVID-19 has spread worldwide - including in Germany [1]. It exerts an unprecedented influence on the daily, professional and leisure life of the entire population and poses extreme challenges to health care systems. As in the whole of Germany, there had been restrictions on public life in Saarland since 16 Mar 2020, which culminated in the public lockdown from the 13th calendar week [1, 2] which ended in the 20th calendar week [1, 3, 4]. Key strategies to contain COVID-19 included an obligation to cover the mouth and nose when visiting health care facilities (from 27 to 2020), the reduction of face-to-face contact by closing public meeting places (e.g. stores and restaurants), curfews and bans on visits to hospital wards [2].

These strategies affect all patients but particularly vulnerable patients such as cancer patiens [5–7] and extend to the whole treatment pathway, from diagnosis [8, 9], through treatments [10, 11], to aftercare [12]. In health care, various attempts were made to maintain diagnostics and treatment quality as much as possible. Nevertheless, there were changes and cancellations of appointments, also in the areas of aftercare as well as psycho-oncology, nutrition and exercise therapies and social counseling [13]. Thus, patients experienced uncertainty in care and were often left with unanswered questions [14], which led them to contact the Cancer Information Service of the German Cancer Research Centre, for example [15]. The number of patients who do not turn to official services despite uncertainty and unanswered questions is unknown. It is well established that confusion, anxiety and insecurity can lead to reduced well-being and even depressive states [16]. In this respect, long-term effects of this burden can be expected months or years later.

Such an unpredictable event as the COVID-19 pandemic may affect study conditions. The present survey investigates the influence of the pandemic on medical care in cancer patients. This supplementary survey was conducted as part of the intervention study “Patient information, communication and competence empowerment in oncology (PIKKO)” [17]. It investigated how patients perceived the PIKKO care services during the pandemic and how patients experienced restrictions due to pandemic containment strategies. We hypothesize that cancer patients in the PIKKO study felt significantly restricted by the COVID-19 strategies, especially those affected by the lockdown (regarding PIKKO). Further, we hypothesize that there was a significant decrease in use of the person-centered intervention modules, i.e., those requiring direct face-to-face contact, such as PN, psycho-social counseling, and courses, while there was no impact on the use of the knowledge database.

## Methods

### Setting – the background of the PIKKO study

PIKKO is a care concept funded by the Innovation Fund of the Federal Joint Committee in Germany (Gemeinsamer Bundesausschuss, funding number 01NVF17011) which supplements oncological care with an additional counseling and information pathway [17]. Participating patients were interviewed multiple times as part of the evaluation of this care concept. The intervention modules, which were available to the patients during the pandemic containment strategies, were: (1) the advice and support provided by a patient navigator (PN), who was available by telephone and in person for all questions concerning the treatment pathway, social law related issues and additional services; (2) a service offered by the Saarland Cancer Society (SCS) consisting of courses (nutritional counseling, art and creative courses, exercise courses, lectures) as well as psychological and psycho-social counseling; (3) a web-based knowledge database (“My PIKKO”) with general and specific information about the respective cancer disease, social law-related information, and additional supporting feature (e.g. a medical dictionary or list of questions to prepare for visits to the doctor) which were quality assured and prepared for laypersons [18]. Patients who were offered the above mentioned PIKKO modules (intervention group, IG) were compared with patients who received treatment as usual (control group, CG). In 2017 PIKKO started with the CG, which was recruited from 1 Nov 2017 to 31 Oct 2018, and surveyed through 31 Oct 2019. The recruitment of IG started on 1 Nov 2018 and ended on 31 Mar 2020, which was exactly in the period of the first lockdown in Germany. Data for the IG were collected through 30 Sep 2020 [17]. The data collection of the CG was not affected by the COVID-19 lockdown, but that of the IG was.

But it was not only the surveys that were affected by the pandemic, but also the intervention itself. The use of PN and offers of the SCS was often linked to a visit to an on-site facility (clinic, practice, course rooms) and thus to movement in the public space. This can be a barrier for many cancer patients because they can no longer participate in the study due to external circumstances - such as lockdown strategies - or they do not want to participate due to their own fears. Fortunately, the PN could also be reached by telephone from the very beginning, so this was already a familiar alternative method of contact for the PIKKO patients. Beginning 30 Mar 2020, the SCS offered its psychological and psycho-social counseling during extended telephone hours and produced course videos to offer the content of the courses to patients online. Other SCS courses took place outdoors (Nordic Walking, QiGong). “My PIKKO” operated independent of location anyway. This meant that the entire intervention was still available.

This supplementary survey, which was not included in the original study design of PIKKO, examines the impact of the pandemic on patients and the use of the intervention modules. Only the IG data collection took place under lockdown conditions and only the IG used the intervention modules. Therefore, all participants in this supplementary survey were from the IG. Participants were already informed about data privacy as part of the PIKKO study (The ethics committee of medical association of the Saarland approved the study protocol on 2 Nov 2017; approval number 114/17. The informed consent by study participants is obtained in a written way.) and the supplementary survey participation was voluntary.

### Participants and inclusion criteria

All participants of the supplementary survey met the inclusion criteria of the PIKKO study (age 18–90 years, diagnosis of any cancer disease, treatment by doctors from the Saarland, insured with one of the four statutory health insurance companies participating in this study) [17], were part of the IG and had completed at least one PIKKO baseline questionnaire.

### Design

In the present survey, we investigated two groups with a quasi experimental design. Group A was not affected (“affected” is related to participation in the PIKKO study) by the lockdown because the patients went through the PIKKO intervention as planned. The end of the PIKKO intervention or the voluntary exit from the PIKKO accompanying survey took place before the lockdown (16 Mar 2020). Group B was affected by the lockdown (in connection with participation in the PIKKO study). Part of the PIKKO intervention and/or survey was conducted during the lockdown, so the end of PIKKO was in or after the lockdown. Group B should still have regular contact (including face-to-face) with the PN, attend (on-site) courses or counseling sessions of the SCS, and complete surveys (which were mailed). All of these may require direct contact, which was limited by the pandemic containment strategies. In addition, as late participants in the PIKKO study, they are still more likely to be in active cancer treatment and are likely to be more frequent guests at medical facilities. Group assignment was not random; rather, but was determined by the timing of inclusion in the PIKKO study (Group A: early inclusion = already enrolled in PIKKO for an average of 358 days at the time of lockdown; Group B: late inclusion = already enrolled in PIKKO for an average of 167 days at the time of lockdown).

The supplementary survey took place from 31 July 2020 to 31 Aug 2020.

### Variables

To assess the impact of pandemic containment strategies on our cancer patients, we asked them questions about four aspects: Restrictions (“Have you had any restrictions with regard to your disease since 16 Mar 2020?: Yes/No If yes, which?”), influence on disease (“Do you think that the limitations due to the COVID-19 pandemic have an influence on the course of your disease?: Yes/No”), use of PIKKO (“Did you use parts of the PIKKO intervention during the limitations due to the COVID-19 pandemic?”), and their own sense of burden (“8 sub-questions on stressful situations to the assessment of the burden of the restrictions (CBS)”).

Our self-designed COVID-19 conditional burden scale (CBS) questions covered the points of (1) hygienic strategies, (2) change of appointments, (3) movement in the public space, (4) cover mouth and nose, (5) no accompanying persons, (6) interaction with the medical staff, (7) interaction with the nursing staff, (8) ban on visits to the wards. Each of the questions could be rated on a 5-point Likert scale (0 = not stressful, 1 = a little stressful, 2 = moderately stressful, 3 = much stressful, 4 = very much stressful). Since not every patient was exposed to all eight stressful situations, the mean should be taken only for the questions answered, so missing (= item did not apply or was not ticked) enters the equation. The following formula was used to compute the CBS score (= the mean of all the patient’s responses): CBS-Score = Sum(CBS1, CBS2, CBS3, CBS4, CBS5, CBS6, CBS7, CBS8) / (8 – Sum(Missing)).

The score ranges from 0 (no load) to 4 (heavy load). Cronbachs Alpha for the cases where all 8 items were completed is 0.9 (N = 52).

Furthermore, previously collected utilization data from the main PIKKO study concerning the intervention modules were analyzed for the COVID-19 period.

### Data sources

The four questions concerning COVID-19 were collected using a two-page questionnaire.

From our regular patient survey [17], socio-demographic and disease data as well as the frequency of PN contacts was taken, retrospective during the last three months before the survey time. The SCS provided data on the use of psychological and psycho-social counseling and of the different courses (Nordic Walking, yoga, QiGong, nutrition, music therapy, art and creativity), which are presented on a monthly basis. A log file of the knowledge database “My PIKKO” allowed to count the monthly accesses of the patients. All frequencies are based on simple counts performed by the patients themselves (contacts to the PN), the SCS (use of their service) or automatically by the website.

### Bias

Since the cancer patients interviewed were already part of the main PIKKO study, a selection bias can be assumed here.

### Study size

A full survey of all living PIKKO-IG participants was intended (n = 503).

### Statistical methods

First, selection effects were investigated by comparing participating and non-participating patients in the supplementary survey. To examine the selection bias, chi-square tests and F-tests were performed (independent variable: patient included versus excluded, depended variables: age, sex, etc.). Next, we compared the both groups of survey participants (not affected, A, and affected, B, by lockdown with regard to PIKKO) regarding to socio-demographic data, disease data, and treatment as well, using chi-square tests and F-tests.

Then we compared both groups (not affected, A, and affected, B, by lockdown with regard to PIKKO) regarding health care related variables.

To examine the restrictions in relation to the disease we conducted chi-square tests.

To quantify the burden due to the pandemic containment strategies, we applied first a linear regression. All single items of the COVID-19 CBS and the sum score of the CBS (in separate calculations) were used as dependent variables. As independent variables we considered Group (0: not affacted, 1: affected), age (grouped by median, 0: under 60 years, 1: 60 + years), gender (0: female, 1: male), children in the household (0: no, 1: yes), financial burden (0: no, 1: yes), period of the most recent illness (dummy variable A: up to 1 year versus 6 + years, dummy variable B: 2–5 years versus 6 + years) and cancer treatment at baseline (dummy variable A: active treatment versus no active treatment, dummy variable B: only rehabilitation versus no active treatment). Based on the estimated regression coefficients we estimated adjusted group means and compared them with t-tests.

The assumption of an influence on the course of the disease and using of parts of the PIKKO intervention during the lockdown was investigated with chi-square tests.

Cramer-V [19] (V > 0.1: small effect; V > 0.3: medium effect; V > 0.5: strong effect) and partial Eta-squared [20] (η²>0.01: small effect; η²>0.06: medium effect; η²>0.14: strong effect) are used as effect sizes.

Missing values did not occur in the dichotomous questions. Missing answers in the CBS that occurred when an item/situation did not apply to the patient were marked as “not applicable” were included in the calculation of the CBS score as “8 - Sum(Missing)”. Only if all subitems were “not applicable”, these cases were excluded from the CBS analysis.

In all analyses, the level of significance was α = 0.05.

Data on the utilization of the PIKKO modules (Patient Navigator, SCS counseling and courses, knowledge database) were analyzed descriptively.

## Results

### Participants

**Fig. 1:**
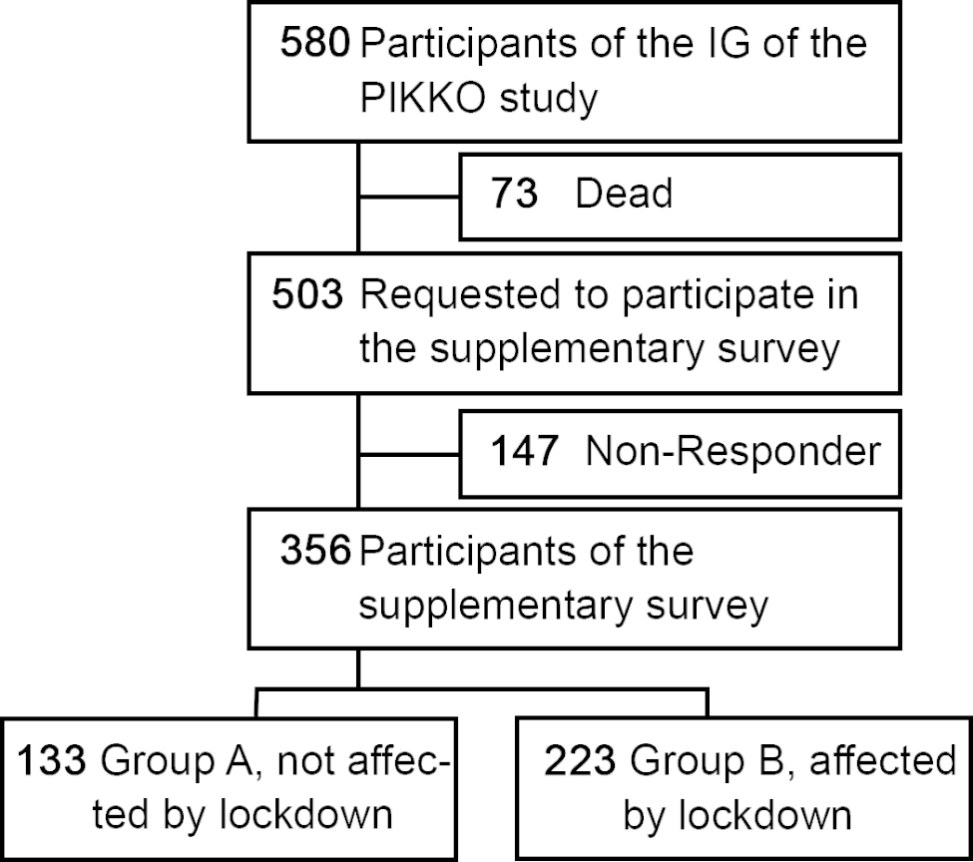
Flow chart of the supplementary survey

A total of 503 (Group A = 241, Group B = 262) patients were contacted (the entire PIKKO intervention group). Of these, n = 356 returned a completed questionnaire of supplementary survey (n_Group A_=133, n_Group B_=223) (Fig. 1). The response rate was therefore 70.8% (356/503).

For unknown reasons 147 patients did not respond and could not be interviewed again in the main PIKKO study (126/147, 85.7%). Others were deceased (9/147, 6.1%), withdrew for health reasons (2/147, 1.4%), or completed the PIKKO surveys but no longer responded (10/147, 6.8%).

### Descriptive data

First, selection effects were investigated, to identify whether the sample of participating (n = 356) and non-participating patients (n = 147) differed. Corresponding statistics are listed in Table 1. The average age (58.7 years, median 59 years) of the participants was significantly higher (small effect). Furthermore, women (72.8%, 259/356) participated more often in the supplementary survey (small effect) and people with children in their own household (21.3%, 76/356) or with financial worries (13.5%, 48/356) participated less often (both, small effects).

**Table 1:** Description of the sample and determination of the differences between the subgroups non-participants and participants as well as groups A and B. Statistical differences (determined by F-test or chi-square test) are marked with asterisks

	Non- participating	Participating supplementary survey		
		Participating	Group A	Group B
Number of patients [n]	147	356	133	223
Age at enrolment [m (sd)]	55.7 (11.1)**	58.7 (10.9)**	57.9 (10.7)	59.2 (11.0)
Age grouped by median				
under 59 years old	54.4% (80/147)	46.9% (167/356)	53.4% (71/133)	43.0% (96/223)
60 + years old	45.6% (67/147)	53.1% (189/356)	46.6% (62/133)	57.0% (127/223)
Gender				
Female	60.5% (89/147)**	72.8% (259/356)**	71.4% (95/133)	73.5% (164/223)
Male	39.5% (58/147)**	27.2% (97/356)**	28.6% (38/133)	26.5% (59/223)
Marital status				
Single	19.0% (28/147)	12.4% (44/356)	12.0% (16/133)	12.6% (28/223)
Married	61.9% (91/147)	68.5% (244/356)	69.9% (93/133)	67.7% (151/223)
Divorced	11.6% (17/147)	11.2% (40/356)	10.5% (14/133)	11.7% (26/223)
Widowed	7.5% (11/147)	7.9% (28/356)	7.5% (10/133)	8.1% (18/223)
Living with the partner	75.9% (110/145)	75.1% (263/350)	72.2% (96/133)	77.0% (167/217)
Children living in the household	31.3% (45/144)*	21.9% (76/347)*	28.3% (36/127)*	18.2% (40/220)*
Financial worries	23.4% (33/141)*	13.6% (48/353)*	19.8% (26/131)**	9.9% (22/222)**
Years of education (school + vocational) [m (sd)]	11.9 (3.2)	12.3 (3.1)	12.4 (3.3)	12.2 (2.9)
School level				
< 10 years of school	49.0% (72/147)	48.3% (172/356)	51.1% (68/133)	46.6% (104/223)
10 years of school	31.3% (46/147)	24.7% (88/356)	18.0% (24/133)	28.7% (64/223)
> 10 years of school	19.7% (29/147)	27.0% (96/356)	30.8% (41/133)	24.7% (55/223)
Period of the most recent illness				
up to 1 year (acute)	85.5% (112/131)	79.5% (268/337)	71.5% (88/123)*	84.1% (180/214)*
2–5 years	9.2% (12/131)	16.0% (54/337)	21.1% (26/123)*	13.1% (28/214)*
> 6 years	5.3% (7/131)	4.5% (15/337)	7.3% (9/123)*	2.8% (6/214)*
Types of cancer grouped				
Gastrointestinal (C00-25)	23.8% (35/147)	18.8% (67/356)	20.3% (27/133)	17.9% (40/223)
Lung and larynx (C32-34)	13.6% (20/147)	9.0% (32/356)	12.0% (16/133)	7.2% (16/223)
Female genitals incl. breast (C50-56)	44.9% (66/147)	53.9% (192/356)	48.1% (64/133)	57.4% (128/223)
Male genitals (C61-62)	6.1% (9/147)	4.2% (15/356)	3.8% (5/133)	4.5% (10/223)
Leukaemia, Lymphoma (C81-96)	9.5% (14/147)	12.4% (44/356)	10.5% (14/133)	13.5% (30/223)
Other	10.9% (16/147)	9.6% (34/356)	13.5% (18/133)	7.2% (16/223)
Cancer attributes				
Tumour spread	47.6% (70/147)	49.4% (176/356)	48.9% (65/133)	49.8% (111/223)
Lymph node metastases	20.4% (30/147)	18.3% (65/356)	21.1% (28/133)	16.6% (37/223)
Distant metastases	15.0% (22/147)	10.7% (38/356)	7.5% (10/133)	12.6% (28/223)
Relapse	2.7% (4/147)	2.5% (9/356)	3.8% (5/133)	1.8% (4/223)
Cancer treatment at baseline				
no active cancer treatment, no ongoing rehabilitation	14.3% (21/147)	17.4% (62/356)	24.1% (32/133)*	13.5% (30/223)*
active cancer treatment	82.3% (121/147)	80.4% (286/356)	72.2% (96/133)*	85.2% (190/223)*
only ongoing rehabilitation	3.4% (5/147)	2.2% (8/356)	3.8% (5/133)*	1.3% (3/223)*

Note: The values are shown for all persons contacted (n = 503), divided into different subgroups: Those who did not respond (n = 147), all participants (n = 356), those who ended PIKKO before lockdown (Group A, n = 133) and those who ended PIKKO in or after lockdown (Group B, n = 223). Statistically significant differences are marked with an asterisk, *p < 0.05, **p < 0.01, ***p < 0.001

The amount of missing data in the burden data varied greatly from item to item: n_missCBS1_=34, n_missCBS2_=148, n_missCBS3_=76, n_missCBS4_=30, n_missCBS5_=119, n_missCBS6_=117, n_missCBS7_=163, n_missCBS8_=251. The large differences can be explained by the different incidence of the situations among the patients.

### Main results: comparison of “not affected” and “affected” by lockdown with regard to PIKKO

Comparisons of group A and group B in terms of socio-demographic data, disease data, and treatment (Table 1) shows that in group B, there were significantly fewer “children living in the household” of the respondents, there were significantly fewer “financial worries”, but there was significantly more often an “acute” cancer disease and there was significantly more often active (surgery, chemotherapy, radiation) treatment at the start of PIKKO (all, small effects).

#### Restrictions in relation to the disease

Out of all participants, 134 (37.6% of the sample) reported any restrictions. Group A and B differed significantly (χ²[1] = 5.177, p = 0.023, OR = 1.694). Of the patients who affected by the lockdown (group B), 42.2% (94/223) reported restrictions (group A = 40/133, 30.1%). In particular, they were significantly more likely to report not using PIKKO modules (52/223, 23.3%; χ²[1] = 5.919, p = 0.015, OR = 2.075) or using them only by telephone (39/223, 17.5%; χ²[1] = 6.977, p = 0.008, OR = 2.607). Group A (16/133, 12.0%) was significantly (χ²[1] = 5.082, p = 0.024, OR = 0.416) more likely to report cancelled appointments than group B (12/223, 5.4%). Rehab cancellations were reported by 8.3% (11/133) of group A and 14.3% (32/223) of group B, with no significant differences between the groups (χ²[1] = 2.899, p = 0.089, OR = 1.858).

#### Burdens due to the pandemic containment strategies

The most perceived burdens of the entire sample were: „Restriction or ban on visits while I was on the ward” (m = 1.92, sd = 1.542); “Wear a protective mouth-nose mask” (m = 1.33, sd = 1.282) and “Restrictions on accompanying persons” (m = 1.19, sd = 1.355). As the analyses show, there were no group differences between group A (not affected) and group B (affected), except for the burden of “restrictions on accompanying persons”. Other factors were more determinant in how strongly a burden is perceived (see Supplementary Material 1 for the results of the linear regression analyses and all group differences and Table 2 for the regression-adjusted means of those factors showing significant differences in some of the items): gender (women were not only more burdened overall, the appointment situation also burdens them more than men); age grouping by median (people under 60 years saw themselves more burdened, especially in the ban on visits to wards, wearing of mouth-nose masks, restrictions on accompanying persons and hygiene strategies); financial burden, even before Corona (people who already felt financially burdened before Corona were also more burdened in the lockdown especially when wearing the mouth-nose masks, moving through public spaces, interacting with medical staff and dealing with hygiene strategies); children in the household (persons with children were more burdened by the appointment situation and the interaction with medical and non-medical personnel).

**Table 2:** Regression-adjusted means, standard deviation, t-statistic and significance (p-value) of the significant factors of the linear regression analyses (see additional file 1) of the burden values

**Group**	**Group A**	**Group B**	**t**	**p-value**
Item 5: Restrictions on accompanying persons	0.773 (0.280)	1.288 (0.300)	2.581	0.011
**Age, grouped by median**	**< 60**	**≥** **60**	**t**	**p-value**
Score	1.195 (0.143)	0.819 (0.149)	-3.850	< 0.001
Item 1: Additional hygiene strategies	0.514 (0.120)	0.335 (0.125)	-2.175	0.030
Item 4: Wear a protective mouth-nose mask	1.910 (0.226)	1.411 (0.235)	-3.242	0.001
Item 5: Restrictions on accompanying persons	1.344 (0.283)	0.718 (0.296)	-3.250	0.001
Item 8: Restriction or ban on visits while I was on the ward	1.065 (0.667)	0.260 (0.696)	-2.384	0.019
**Gender**	**female**	**male**	**t**	**p-value**
Score	1.128 (0.135)	0.886 (0.159)	-2.315	0.021
Item 2: The changed appointment situation	1.255 (0.221)	0.804 (0.273)	-2.366	0.019
**Children in the household**	**yes**	**no**	**t**	**p-value**
Item 2: The changed appointment situation	1.232 (0.273)	0.827 (0.227)	1.993	0.048
Item 6: Changes in the way medical staff interact	1.287 (0.269)	0.809 (0.225)	2.398	0.017
Item 7: Changes in the way care staff interact	1.201 (0.322)	0.741 (0.276)	2.060	0.041
**Financial burden**	**yes**	**no**	**t**	**p-value**
Score	1.243 (0.167)	0.771 (0.136)	3.655	< 0.001
Item 1: Additional hygiene strategies	0.676 (0.140)	0.173 (0.113)	4.622	< 0.001
Item 3: Moving to appointments in public	1.317 (0.248)	0.774 (0.198)	2.807	0.005
Item 4: Wear a protective mouth-nose mask	2.058 (0.264)	1.263 (0.214)	3.878	< 0.001
Item 6: Changes in the way medical staff interact	1.275 (0.267)	0.821 (0.229)	2.229	0.027

#### Assumption of an influence on the course of the disease

Although 39% (140/356) said they fear that the restrictions will have an influence on the course of their disease, in most cases a psychological influence (124/140, 88.6%). The two groups did not differ on this point (χ²[1] = 0.024, p = 0.876, Cramers V = 0.008).

#### Using of parts of the PIKKO intervention during the lockdown

55.2% of the participants in group B (123/223) and 36.8% in group A (49/133) reported to the additional survey that they had used some PIKKO moduls during the lockdown. The two groups differed significantly on this point (χ²[1] = 11.191, p = 0.001, Cramers V = 0.177).

### Other analyses: general use of the PIKKO modules during the lockdown

Beyond the information from the supplementary survey, the data from the regular patient questionnaires (contacts to PNs), the SCS data and the logfile data from the knowledge database provide information about the use of the PIKKO modules during the lockdown.

**Fig. 2:**
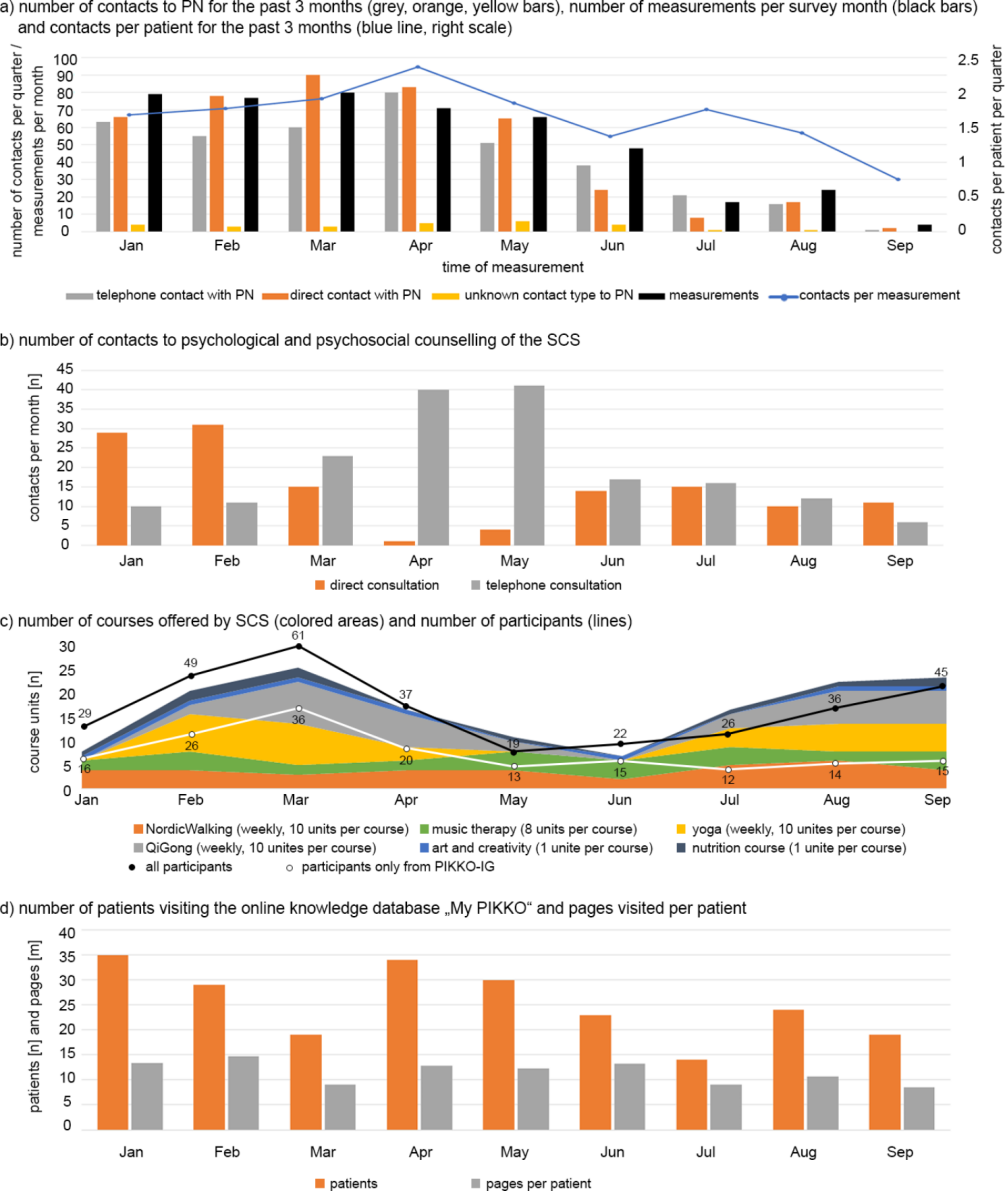
The utilization of the PIKKO-intervention-moduls (**a**) patient navigator (PN), (**b**) psychological and psycho-social counseling of the Saarland Cancer Society (SCS), (**c**) patient courses of the SCS and (**d**) knowledge database „My PIKKO“ in 2020. Either the numbers (n) or the mean values (m) are given. The monthly data in part a) are to be considered as measurement time points; the questions to patients about the number of contacts referred to the last 3 months

The contacts to the PNs are shown in Fig. 2a. Patient quarterly contacts to the PN initially increased slightly at the beginning of the lockdown. The March and April measurements primarily had higher quarterly values for live contacts (patients who were still inpatients and could meet the PN directly in the clinic). In April, there was a significant increase in telephone contacts. The decrease from May can be explained by the decrease in measurements (black bar; patients send back fewer questionnaires). The average number of contacts per patient per quarter (blue line) also increased through April to 2.37 (2019 average was 2.22, January through March 2020 average was 1.79) before also decreasing due to fewer measurements.

Utilization of SCS psychological psycho-social counseling is shown in Fig. 2b. It is noticeable that there were significantly fewer direct consultations in the months of the lockdown (March to May), with the minimum of 1 in April. Instead, the telephone service was expanded and used much more frequently.

As with counseling, both the number of offerings (colored areas) and the number of participants (lines) for courses (Fig. 2c) declined during the lockdown. Especially outdoor offers (Nordic Walking, QiGong) took place. Yoga paused in May and June, QiGong paused in June, after the lockdown. Nutrition courses were not offered in April and June. There were no art and creativity courses in May and July.

The knowledge database (see Fig. 2d) has been continuously used by an average of 25 patients per month, which is slightly less than the average of 34 monthly users in 2019. However, this is due more to the end of the project in September 2020 and the declining number of participants by then than to the pandemic. The months of the lockdown, April and May, had an above-average number of users. On average, a patient viewed 12 pages (13 were in 2019) of the knowledge database per month.

## Discussion

It was the aim of this supplementary survey to identify the restrictions and burdens of the pandemic containment strategies on the PIKKO cancer patients and thus on the PIKKO study itself.

To this aim, we surveyed participants and analyzed these data using frequency analyses and linear regression analyses to determine which factors more strongly influenced patients’ perceived burden.

Our first hypothesis that cancer patients in the PIKKO study felt restricted by the COVID-19 strategies, especially those affected by the lockdown (regarding PIKKO), can be confirmed. Fears of an impact on recovery were expected by the patients, which was similarly found by the online survey of the PRIO Working Group [16]. Mental health problems were expected to be the most common in both studies. Regarding the degree of burden (measured with the CBS), the group distinction of affected/not affected by the lockdown was not significant. Other factors were significant: gender (women reported more burden), age (people under 60 saw themselves as more burdened) or pre-existing areas of burden (money and children) had an amplifying effect on this stress situation.

Two of the greatest burdens of the COVID-19 lockdown were posed by the „ban on visits to the wards“ and „no accompanying persons“, two conditions that were a great imposition for patients with life-threatening diseases such as cancer with an increased need for assistance [16] .

Burdens due to change of appointments were also reported. Even though Fröhling et al. [13] stated that therapies were not or only slightly affected by the pandemic restrictions, uncertainties and questions still arise among patients [14, 15]. After all, treatment delays may be associated with higher mortality [21]. Furthermore, the most obvious of all strategies, „cover mouth and nose“, increased the feeling of suffering restrictions, especially when a patient has to be treated repeatedly in medical facilities. This pandemic protection measure could even lead to hypercapnia in patients with impaired lung function [22] and thus to further problems.

The second hypothesis, that the restrictions caused a decrease in the use of person-centered intervention modules, can only be partially confirmed. The data showed that PN, as well as the SCS counseling and course service and the knowledge database, continued to be used, but in an adapted form. Briefly at the beginning of the lockdown, there were more contacts to the PN, especially live contacts. Many PNs were employed in clinics. This presumably responded to the ban on visits to the ward for inpatients. SCS also reported significantly more telephone consultations during the months of the lockdown. The SCS expanded this offer when face-to-face contact was restricted. Some offerings, such as nutrition courses, were limited, while outdoor Nordic Walking was offered continuously. These types of restrictions were also reported by Fröhling et al. in cancer care centers, in which there were restrictions on psycho-oncology, nutrition and exercise therapies, and social counseling for up to 12 weeks [13]. The frequency of the knowledge database use, on the other hand, was only slightly affected by the pandemic.

The present survey revealed that the pandemic had negative effects on the medical care of cancer patients. Supportive offers such as counseling by PNs in clinics or by SCS counselors via phone, as well as adapting and maintaining other services such as exercise programs, are possible and necessary to mitigate the negative aspects of the pandemic restrictions. Others also reported good uptake for new telepsychological services during pandemic periods [7, 23] or established similar online concepts such as telerehabilitation, which were well received by patients [24]. Such online services can support cancer patients in times of crisis [7]. This is in line with the EU Commission’s new anti-cancer plan, which aims to strengthen telemedicine and remote treatment via computer [25]. For medical care, the study provides evidence that support services for cancer patients, such as psychological and psycho-social counseling and information services, if adapted quickly to distance care, are relatively robust to disease containment strategies such as a lockdown. This can provide additional support to cancer patients even in times of crisis.

## Strengths of the study

We were able to provide insight into how an unforeseen disruption such as the COVID-19 crisis can impact an intervention study. This showed that a combination of flexibility (e.g., switching courses), diversity (e.g., face-to-face opportunities for patients), and location independence (e.g., web-based knowledge base) ensures that offerings for patients (here, cancer patients) can be maintained even when much else is no longer possible.

Furthermore, it became apparent that burdens felt in addition to the disease (due to containment strategies and fears and worries) are not perceived with the same intensity by all groups of people, and that socio-demographic factors may be more decisive for this perception.

### Limitations

Data on contacts to the PN are based on patient self-reports, some of whom were undergoing cancer treatment. There is evidence that chemotherapy can impair memory and recall [26], which is why information on the number of telephone calls with the PN, for example, could be inaccurate.

The supplementary survey took place in the summer of 2020, a few weeks after the first lockdown. Memories of something that happened a few weeks prior could be distorted as described above.

The sample of this supplementary survey is not representative of the PIKKO intervention group in all respects. For example, patients were more likely not to participate if they were still working, younger, had children in the household, or reported financial worries before the pandemic.

## Electronic supplementary material

Below is the link to the electronic supplementary material.

Supplementary Material 1

## Data Availability

The datasets used and/or analysed during the current study available from the corresponding author on reasonable request.

## Abbreviations

CBS: COVID-19 conditional burden scale
CG: control group
IG: intervention group
ODB: online knowledge database
PIKKO: Patient information, communication and competence empowerment in oncology
PN: patient navigator (PN)
SCS: Saarland Cancer Society
