# Supplementary material for: The impact of the COVID-19 pandemic restrictions on the health care utilization of cancer patients
Source: BMC Cancer. 2023 May 15;23:439. doi: 10.1186/s12885-023-10945-9 (PMC10183678; doi:10.1186/s12885-023-10945-9)
Supplement: Supplementary file 1 — Supplementary Material 1 [file 12885_2023_10945_MOESM1_ESM.docx]

Additional Table 1 Linear regression analyses of the burden items as dependent variables and with the differences identified in the group comparison as independent variables.

|  | **Score** | **Item 1** | **Item 2** | **Item 3** | **Item 4** | **Item 5** | **Item 6** | **Item 7** | **Item 8** |
| --- | --- | --- | --- | --- | --- | --- | --- | --- | --- |
|  | **b (SE)** | **b (SE)** | **b (SE)** | **b (SE)** | **b (SE)** | **b (SE)** | **b (SE)** | **b (SE)** | **b (SE)** |
| Intercept | 1.120  (0.265) | 0.357  (0.222) | 1.135  (0.481) | 1.511  (0.385) | 2.020  (0.417) | 1.225  (0.520) | 0.582  (0.407) | 0.686  (0.496) | 0.440  (1.558) |
| Group | 0.097  (0.098) | -0.125  (0.082) | 0.181  (0.177) | 0.036  (0.153) | -0.043  (0.155) | **0.515***  **(0.200)** | 0.163  (0.165) | 0.256  (0.183) | 0.619  (0.344) |
| Age | **-0.375*****  **(0.098)** | **-0.179***  **(0.082)** | -0.338  (0.176) | -0.266  (0.151) | **-0.499****  **(0.154)** | **-0.626****  **(0.192)** | -0.211  (0.164) | -0.228  (0.183) | **-0.805***  **(0.338)** |
| Gender | **-0.241***  **(0.104)** | -0.071  (0.088) | **-0.450***  **(0.190)** | -0.238  (0.161) | -0.322  (0.164) | -0.298  (0.212) | -0.185  (0.171) | -0.213  (0.185) | -0.190  (0.361) |
| Children in the household | 0.126  (0.120) | -0.002  (0.100) | **0.405***  **(0.203)** | 0.206  (0.184) | -0.071  (0.189) | -0.036  (0.225) | **0.479***  **(0.200)** | **0.459***  **(0.223)** | -0.058  (0.401) |
| Financial burden | **0.472*****  **(0.129)** | **0.503*****  **(0.109)** | 0.153  (0.217) | **0.543****  **(0.194)** | **0.796*****  **(0.205)** | 0.404  (0.251) | **0.454***  **(0.204)** | 0.367  (0.214) | 0.438  (0.398) |
| Period of the most recent illness | | | | | | | | | |
| Dummy variable A | -0.174  (0.241) | -0.027  (0.201) | -0.081  (0.432) | -0.515  (0.347) | -0.473  (0.380) | -0.353  (0.472) | 0.101  (0.367) | -0.221  (0.466) | 1.158  (1.524) |
| Dummy variable B | -0.160  (0.259) | 0.075  (0.216) | -0.313  (0.466) | -0.548  (0.379) | -0.180  (0.408) | -0.385  (0.509) | 0.167  (0.399) | -0.477  (0.497) | 0.377  (1.600) |
| Cancer treatment at baseline | | | | | | | | | |
| Dummy variable A | 0.065 (0.126) | 0.093  (0.106) | -0.070  (0.239) | -0.039  (0.189) | 0.030  (0.199) | 0.365  (0.248) | 0.005  (0.206) | 0.205  (0.236) | 0.442  (0.388) |
| Dummy variable B | -0.189  (0.298) | -0.131  (0.249) | 0.222  (0.472) | -0.718  (0.430) | -0.245  (0.469) | -0.151  (0.576) | 0.075  (0.531) | 0.385  (0.676) | -1.314  (1.103) |

*Note: Score=Score of the COVID-19 conditional burden scale Item 1=Additional hygiene strategies; Item 2=The changed appointment situation; Item 3=Moving to appointments in public; Item 4=Wear a protective mouth-nose mask; Item 5=Restrictions on accompanying persons; Item 6=Changes in the way medical staff interact; Item 7=Changes in the way care staff interact; Item 8=Restriction or ban on visits while I was on the ward.* *Significance is indicates by an asterisk, *p<0.05, **p<0.01, ***p<0.001. b… unstandardizied regression coefficient, SE…standard error. Period of the most recent illness: dummy variable A=up to 1 year versus 6+ years, dummy variable B=2-5 years versus 6+ years. Cancer treatment at baseline: dummy variable A=active treatment versus no active treatment, dummy variable B=only rehabilitation versus no active treatment.*
